# A short form of the Crisis in Family Systems (CRISYS) in a racially diverse sample of pregnant women

**DOI:** 10.1007/s12144-021-02335-w

**Published:** 2021-10-01

**Authors:** Phillip Sherlock, Madeleine U. Shalowitz, Carolyn Berry, David Cella, Courtney K. Blackwell, Whitney Cowell, Karen M. Reyes Rodriguez, Rosalind J. Wright

**Affiliations:** 1 Northwestern University Feinberg School of Medicine, Chicago, IL, USA; 2 Rush University Medical Center, Chicago, IL, USA; 3 NYU Grossman School of Medicine, New York, NY, USA; 4 Icahn School of Medicine at Mount Sinai, New York, NY, USA

**Keywords:** Life-event checklist, CRISYS-short form, CRISYS-SF, Social Determinants of Health, Stressful Life Events, Latent Class Analysis

## Abstract

Assessing stressful life events in large-scale epidemiologic studies is challenged by the need to measure potential stressful events in a reasonably comprehensible manner balanced with burden on participants and research staff. The aim of this paper was to create a short form of the Crisis in Family Systems-Revised (CRISYS-R) plus 17 acculturation items, a measure that captures contemporary life stressors across 11 domains. Latent class analysis (LCA) was used to segment the sample of 884 women from the PRogramming of Intergenerational Stress Mechanisms (PRISM) study experiencing different patterns of exposure to stressful events and identify items from each domain that best discriminate between individuals with different patterns of stressful-event exposures (high vs. low stress exposure). The results from the LCA, in conjunction with the expert opinions provided by the original developers of the CRISYS, yielded a 24-item item short form (CRISYS-SF) with at least one question from each of the original domains. Scores on the 24-item CRISYS-SF had high correlations with scores on the 80-item CRISYS.

## Introduction

Stress can be conceptualized and measured in different ways depending upon the research goals (Schreier et al., 2011). Stress, from a variety of sources, can have a significant impact on the psychological, social, and physical health of individuals (Cohen et al., 2007). A common approach to broadly measuring the sources of stress in larger-scale epidemiologic studies is based in the environmental stress perspective, which is focused on external demands typically encountered as part of one’s life experience. Life-event checklists are widely used to assess and sum the types of stressors experienced by a population within a given timeframe (Turner and Wheaton, 1995). However, recent studies have begun to investigate differences between using sum scores, individual stressors, and latent class approaches to understand relationships between stressful events and health outcomes (Heidinger & Willson, 2019; Lacey et al., 2020; Ziobrowski et al., 2020). Nevertheless, a large body of literature supports a link between life events and health outcomes (Cohen et al., 2019) and the utility of checklists in larger epidemiologic samples as they are efficient, inexpensive, and do not require special training to administer. The more comprehensively the checklist covers events across a number of life domains, it is likely to be a reasonable representation of events experienced across the population of interest. However, the length of the questionnaire must also be balanced with the potential to overburden the research subjects. This is particularly true in studies that have an extensive battery of questionnaires to administer in addition to those focused on stress. Factors including questionnaire length contribute to respondent burden and survey fatigue, which can contribute to reduced response rate, incomplete data, and consequent reduced data quality in epidemiologic studies (Rolstad et al., 2011).

The Crisis in Family Systems-Revised (CRISYS-R) (Berry et al., 2001) was developed as a more contemporary measure of life events with reduced cultural and socioeconomic biases that would be more broadly applicable across US populations (Shalowitz et al., 1998). The originally published CRISYS-R includes 63 items from 11 content domains (Shalowitz et al., 1998). These 11 domains encompass financial; legal; relationship; medical issues pertaining to one’s self; medical issues pertaining to others; community safety; safety in the home; housing; career; prejudice; and authority. Subsequently, as acculturation stress emerged as an important construct in the context of health disparities (Fox et al., 2017), 17 additional sub-items related to acculturation were included in the measure to expand coverage of this important domain. While the comprehensive scope of more contemporary life events covered by the CRISYS-R plus these acculturation items is a noted strength, the length of the survey (now 80 items) has limited its wide scale adaptation in epidemiologic research. This motivated the current study, which used empirical, model-based inquiry and expert consensus in order to create a short form for the 80-item CRISYS, which includes 17 acculturation, while maintaining the breadth of domains covered including the acculturation items.

The field of life course research has enhanced our understanding of the relative importance of exposures during different life stages in relation to health trajectories (Ben-Shlomo et al., 2016). Across the lifespan, exposure to stressful events in critical windows can have more significant and lasting effects on health than exposures outside these windows (Cohen et al., 2019). There has been particular focus on the in utero period in relation to chronic disease programming in offspring (Gómez-Roig et al., 2021) with more recent recognition that pregnancy is a critical window during which exposures can impact women’s long-term health more significantly than if they are experienced in other life stages (Varshavsky et al., 2020; Wright, 2021). Thus, refining stress assessment measures in pregnant samples is an important area of research.

In the current study, we leveraged data from an ongoing birth cohort of pregnant women based in the northeastern United States to create a short form by synergizing empirical, model-based inquiry based on latent class analysis (LCA) and expert consensus, while maintaining the breadth of domains covered by the foundational, 80-item CRISYS measure as well as the acculturation items. Specifically, the goal of LCA is to find the mixture that optimally differentiates between subgroups of people, whereby the homogeneity of experiences within class and the heterogeneity of experiences across classes are maximized. Based on this understanding of the independence of life stressors and the occurrence of qualitatively distinct constellations of stressful events, the purpose of this paper is to create a short form of the 80-item CRISYS (CRISYS-SF) using LCA. Previous work on developing a short form for the Facebook Addiction Test demonstrated the utility of using LCA as a model-based criterion for investigating sources of variance in multifaceted constructs that are usually summed to a single score (Dantlgraber et al., 2016). The current study utilized LCA in order to determine the greatest sources of variation with respect to stressors across subgroups of respondents, choosing items that demonstrated the greatest difference in expected endorsement probabilities. Please see S1 Appendix *for* the full list of 80 items used as a starting point in the creation of the short form of the CRISYS.

Although the CRISYS-SF will necessarily have fewer items than the 80-item CRISYS, the goal was to retain items that have the greatest differences in their endorsement probabilities across groups. Furthermore, this research team aimed to maintain the breadth of the CRISYS by retaining at least one item from each of the domains and balancing the results of the LCA with the expert opinions of the CRISYS developers. Therefore, items were not chosen for the final short form based simply on their occurrence frequencies, but rather the difference in endorsement probabilities by domain. This balanced approach is in line with recommendations for balancing empirical results and fit indices with substantive theory and model utility (Masyn, 2013), albeit not in the traditional sense of enumeration, but with regard to guiding the interpretation of latent class model results with substantive theory and regard for utility.

## Method

### Sample

The PRogramming of Intergenerational Stress Mechanisms (PRISM) study is an ongoing prospective pregnancy cohort that recruited women from prenatal clinics in New York City and Boston beginning in 2011. At the time of this analysis, 1091 pregnant women had been enrolled; we excluded 207 women that did not complete the CRISYS, resulting in an analytic sample of 884 women. The 63-item CRISYS-R, as well as the additional 17 acculturation items, was completed during pregnancy in a face-to-face interview (96.5%) or by telephone (3.5%) in their preferred language (English: 83.6%, Spanish: 16.4%); if a woman completed the CRISYS during more than one pregnancy, only the first response was considered. The sample is socioeconomically and demographically diverse with approximately 20%, 40% and 30% of women self-reporting White/non-Hispanic, Black/Black-Hispanic, and Hispanic/non-Black race/ethnicity, respectively (Table 1). Nearly one-third of women were born outside of the United States and 20% reported primarily speaking Spanish or both Spanish and English in the home. Approximately 20% of women had less than a high school education and nearly 60% reported not having enough money (16%) or just enough money (44%) at the end of the month. The mean age of participants was 29.5 years (range: 18.3 – 49.4 years). Additional characteristics of the study sample are provided in Table 1. All study procedures were approved by the institutional review boards at the Icahn School of Medicine at Mount Sinai in New York City or the Brigham and Women’s Hospital in Boston. Written informed consent was obtained from all participants in their primary language.

### Latent Class Analysis, Item Reduction, and Validation

Using all of the CRISYS-R items as well as 17 items related to acculturation, a series of latent class models were estimated. Enumeration (i.e., the selection of the optimal number of *k* classes) was based on empirical fit indices/tests and the overall utility of the model in distinguishing between items that differentiate between high and low stress individuals within domain. In accordance with insights from Tein et al. (2013), several fit indices were used in the enumeration process because no one fit index always produces the largest statistical power. Furthermore, the statistical power of fit indices depends on the number of indicators and sample size. The empirical indices used in enumeration include the following: Bayesian Information Criterion (BIC) (Schwarz, 1978), Lo–Mendell–Rubin Likelihood Ratio Test (LMR; Lo et al., 2001), Vuong-Lo-Mendell-Rubin Likelihood Ratio Test (VLMR; Lo et al., 2001), Parametric Bootstrapped Likelihood Ratio Test (BLRT; McLachlan & Peel, 2000), and class proportions (i.e., the percentages of respondents in each latent class). In selecting the final model with *K* classes, the model with the lowest BIC was considered to have evidence of the best relative fit. The LMR, VLMR, and BLRT are interpreted such that a significant statistic implies the model with *k* + 1 classes provides significantly better fit compared to the model with *k* classes. A non-significant LMR, VLMRT, or BLRT statistic fails to reject the null hypothesis of model equivalence, such that one would fail to provide evidence that the model with *k* + 1 classes has better fit than the *k*-class model. Related to enumeration based on class proportions, previous research has pointed toward the difficulty of capturing rare latent classes (i.e., classes representing 1–8% of the population) especially when there are a large number of indicators relative to the total sample size (Masyn, 2013). Therefore, LCA solutions with classes containing 1–8% of the sample were discarded, as it is likely these solutions contained spurious classes. Furthermore, classification quality was evaluated using entropy and mean posterior probabilities based on most likely class membership to determine the optimal latent class solution.

After enumeration, the items were ordered by domain and the differences in the endorsement probabilities between individuals across the *k* classes (i.e., discrimination). The original aim was to retain the two items from each domain with the greatest discrimination values. After the initial evaluation of the model-based approach, items were added or removed based on expert consensus as well as consideration of the discrimination values for items that were not in the top two of their respective domains. Reliability of the CRISYS-SF was evaluated using α (Cronbach, 1951). Although the use of LCA would seem to be at odds with reporting Cronbach’s α, which assumes unidimensionality, LCA was only used to prune items from the CRISYS. The question of the optimal use of responses to the CRISYS-SF (i.e., stressor sum score vs. individual items vs. latent class regression) in future studies will be study dependent, as it is not possible to understand how the CRISYS-SF will associate with particular outcomes without testing differences between predictor formulations. The final CRISYS-SF was evaluated by calculating the Spearman correlation between the total score of the CRISYS and the CRISYS-SF, with a high correlation indicating that the LCA approach provided a succinct way to capture the group of items that best approximate the variation in stressful experiences provided by the full version. This approach was used to determine correspondence between CRISYS and CRISYS-SF scores after significant item reduction.

### Expert Panel Review

The expert panel has over 20 years of experience using the CRISYS-R with and without the acculturation items. They recognize the need to offer a shortened version for screening and to include as a construct in larger models, rather than to identify a broad range of items that would guide intervention. The original designers of the instrument (MUS and CAB) were invited to be authors on the manuscript so that they would contribute to the background, the expert validation of retained/dropped items and the interpretation of the results. Co-Author Reyes was involved in the development of the acculturation items and has years of personal experience administering the questionnaire to participants. Co-Author Wright was among the first scientists to incorporate the CRISYS in her longitudinal studies of stress and co-chaired an NHLBI expert panel on measurement that recommended the CRISYS to measure stress in studies of asthma.

## Results

### Latent Class Enumeration

The enumeration process based on the BIC, LMR, VLMRT, BLRT, and class proportions resulted in the selection of a two-class model being chosen as having the best fit relative to the one- and three-class model. Table 2 contains the fit indices used in enumeration. The two-class model demonstrated superior relative fit compared to the one-class model based on having a lower BIC value. Although the BIC value for the three-class model was about 11 points less than the BIC for the two-class model, further consideration was given to the two-class model based on the LMR, VLMR, BLR, and class proportions. The significant LMR and VLMR tests between the one- and two-class models indicated that the two-class model provides significantly better fit. The non-significant LMR and VLMR tests of the three-class model indicated that the two-class model does not fit the data significantly worse than the three-class model. Thus, the LMR and VLMR test supported the superior fit of the two-class model, relative to the one- and three-class models. While the BLRT between the one- and two-class models pointed to the superior fit of the two-class model, the BLRT between the two- and three-class models with 1500 initial stage random starts and 750 final stage optimizations resulted in a non-trustworthy p-value due to local maxima. Furthermore, the two-class solution contained two classes with 35% and 65% of the sample, respectively. The three-class model contained three classes with 56%, 38%, and 5% of the sample, respectively. Therefore, the three-class model is likely to contain a spurious class. Furthermore, the entropies of the two- and three-class models are nearly identical and the mean posterior probabilities based on most likely group membership were very similar, although they were slightly higher in the two-class solution. In the end, based on the evidence provided by the BIC, aBIC, LMR, VLMR, BLR, class proportions, and classification quality, the two-class solution was chosen as the optimal model.

### Final LCA Results and Item Selection

Table 3 includes the CRISYS domains and their respective items, as well as the expected endorsement probabilities from the final two classes, the discrimination (i.e., the difference in the item probabilities between the two classes), the expert review (i.e., added or removed), and whether the item was included in the final short form. After selecting the items based on the discriminations with the original domains as a guide, the expert panel removed three items and added five items. *Did you have trouble with social service agencies?* was not retained because the expert panel believed, based on experience administering the measure that there was confusion among participants of which agencies this entailed. *Did your income decrease by a lot?* and *Did you go without food because you didn’t have the money to pay for it?* were added despite not having the highest within-domain discrimination values as there was consensus that direct questions related to income changes and food insecurity would be relevant for future health disparities research. *Did anyone in your family go to jail?* was dropped because *Did anyone in your family get arrested?* was retained and the expert panel believed there was ambiguity between jail and prison, being arrested was more direct, and retaining both of these items would have been redundant*—Was anyone in your family pulled over or questioned by the police?* was then added as this was felt to be a stressor, particularly to certain racial/ethnic groups regardless of whether this resulted in an arrest. *Did a family member die?* was added by the expert panel because this event is included on the majority of life event measures. *Did your regular childcare arrangements change in any way?* was added because this was deemed an important topic unrelated to any of the retained items. *Were you a victim of a crime while you were in your home?* was dropped because more specific questions related to neighborhood violence and emotional/physical abuse had already been retained. The modifications in conjunction with the LCA resulted in a 24-item CRISYS-SF, which included at least one item from each of the original 11 domains.

S1 Figure displays the expected endorsement rates by latent class for the 24-item short form. The expected endorsement rates demonstrate clear separation in endorsement probabilities across all of the items. Cronbach’s α for the CRISYS-SF was equal to 0.70. This α level is less than the preferred 0.8 threshold and may be an indication that future studies should explore latent class regression rather than sum scores, as an approach to understanding the relationships between stressful experiences and health outcomes. The total score of the CRISYS-SF had a Spearman correlation with the total score from the CRISYS equal to 0.89 for the entire sample. The Spearman correlation between the total scores from the CRISYS and the CRISYS-SF for individuals who received the Spanish and English versions of the CRISYS were 0.87 and 0.90, respectively. Furthermore, the correlation between the total scores from the CRISYS and the CRISYS-SF for individuals who completed at least a college degree and those who did not were 0.82 and 0.89, respectively. Although, Spanish vs. English speakers and college degree vs. no college degree, represent only two possibilities for examining the correlations between the CRISYS and CRISYS-SF the results provide evidence that the short form does not result in drastically different information across two common covariates in health research—language and education.

S1 Fig. Expected endorsement probabilities for the CRISYS-SF items chosen from the final latent class model.

## Discussion

Factors including questionnaire length contribute to respondent burden and can contribute to reduced response rate, incomplete data, and consequent reduced data quality in epidemiologic studies (Rolstad et al., 2011). Response burden may be particularly problematic in larger scale epidemiologic studies administering a large battery of questionnaires. The results of this study provide support for the validity of the CRISYS-SF, a short form of the 80-item CRISYS-R including acculturation items. LCA, a categorical latent variable approach, was used to first segment the sample of individuals experiencing different patterns of exposure to stressful events.

In the current study, empirical, model-based inquiry and expert consensus were synergized in order to create a short form for the CRISYS, while maintaining the breadth of domains covered by the foundational measure as well as the acculturation items. The challenge encountered at the outset of this study is best characterized by the difference between life stressors and items in a continuous latent trait inventory (i.e., depression). Although groups of individuals may experience several life stressors captured within a particular CRISYS domain, items within a domain are best thought of as capturing qualitatively distinct stressors rather than constituting a scale. The distinction between a typical group of scales, which would be analyzed with common continuous latent trait methods (e.g., confirmatory factor analysis, item response models, etc.), and the CRISYS domains is typified by the theoretical independence of the stressors. Life stressors, unlike items in a depression or anxiety inventory, are not captured by an underlying continuous latent variable (i.e., ability), which has associated assumptions like local independence and monotonicity, whereby items are independent of one another given the individual’s score on the latent variable and the probability of endorsing an item increases as a function of the underlying latent trait and is related to the item parameters (Reckase, 2009). These item-level assumptions are potentially unrealistic for a life stressor inventory like the CRISYS, which covers a wide-range of life stressors. Furthermore, these stressors that may be similar, but qualitatively unrelated in a way that would comply with or lend to the fulfillment of assumptions like local independence and monotonicity because individuals with higher total scores may simply not have experienced some life stressors that had higher rates of endorsement (i.e., low item difficulty). Rather, the CRISYS items were modeled with a categorical latent variable (e.g., latent class analysis; LCA). The current study utilized LCA in order to determine the greatest sources of variation with respect to stressors across subgroups of respondents, choosing items that best differentiate between subgroups. This was accomplished by first identifying the two-class solution as the optimal mixture based on all of the original life stressors from the CRISYS. Finally, an expert panel was convened to select the final short form items that included items that occurred most commonly in the higher life-stressor group and less commonly in the lower life-stressor group.

The optimal latent class solution was obtained by segmenting the original sample into two subgroups (i.e., high- and low-stress classes). Following enumeration, items were considered for inclusion in the CRISYS-SF based on their LCA discrimination values (i.e., the difference in expected endorsement probabilities between the latent classes). The results from the LCA, in conjunction with the expert opinions provided by the authors of this paper, yielded a 24-item item short form with acceptable reliability. The total scores for the CRISYS-SF had a Spearman correlation with the total scores of the CRISYS equal to 0.89. This provided evidence of the robustness of the CRISYS-SF, which included only 30% of the original items in the CRISYS. Furthermore, each of the domains was represented by at least one item, which extends the breadth and integrity of the CRISYS to the CRISYS-SF. As noted before, the construction of this short form or any life stressor inventory is particularly challenging because stressful life events do not beget fulfillment of item-level assumptions related to continuous underlying latent variable modeling. This study involved to the use of LCA as a method for pruning the CIRSYS, but researchers will likely find different latent class solutions with subsequent latent class models using the final CRISYS-SF items. Therefore, researchers interested in in using the CRISYS-SF to investigate relationships between stressful life events and health outcomes are encouraged to explore differences in using cumulative risks represented by sum scores as predictors versus latent class regression.

Development of a valid and reliable abbreviated survey to assess stressful life events has other implications. The evidence of a relationship between stress and health has led to a relatively recent awakening of interest among health care professionals and health care financing entities. A growing consensus is gaining traction for health care providers to systematically identify and then intervene in sources of stress (collectively termed “social determinants of health”, SDoH) to improve health or prevent disease. A recent position paper of the US Preventive Services Task Force calls for “valid and reliable screening tools” for use in primary care and evidence that “screening…lead[s] to reduced morbidity, mortality or both from relevant health conditions (Davidson et al., 2020). Proposed screeners exist that contain individual items that are known to impact health, and these items pick up needs one-by-one for a given individual. While this individual approach has merit, largely these screening tools have not been assessed for psychometric properties as a validated instrument to identify high risk populations with complex needs that act together to have an impact on health. This paper demonstrates the type of statistical approach necessary to have a valid, reliable and reproducible way to capture SDoH information that relates directly to biological mechanisms affecting health while, at the same time, highlighting opportunities for intervention. The CRISYS-SF offers promise as a way to screen for SDoH in a clinical setting, identify resources for individual areas of stress, and refer individuals for targeted intervention. Further, the CRISYS-SF can be used to investigate statistical relationships between SDoH and adverse health conditions over time, and the impact of interventions.

## Limitations and Future Directions

This study involved a single cohort entirely composed of women but as noted in the introduction, pregnancy is a critical window for heightened vulnerability to toxins including stress for both maternal and child health. However, each of the items retained in the CRISYS-SF could be answered by anyone, regardless of sex. Future researchers interested in using the CRISYS-SF to understand the effects of stressful life events on health outcomes are encouraged to investigate possible sex differences in item response frequency, distribution, and predictive value.

## Supplementary Material

1804733_Sup_File_1

1804733_Sup_File_2

## Figures and Tables

**Table 1:** Sociodemographic characteristics of the PRISM sample (n = 884)

	N (%)
Age	
< 20 years	36 (4.1)
20–30 years	428 (48.4)
31–40 years	386 (43.7)
> 40 years	34 (3.8)
Race/ethnicity	
White, non-Hispanic	167 (18.9)
Black/Black-Hispanic	374 (42.3)
Hispanic, non-Black	289 (32.7)
Other	45 (5.1)
Missing	9 (1.0)
Language spoken in home	
English	673 (76.1)
Spanish	114 (12.9)
English & Spanish	68 (7.7)
Missing	29 (3.3)
Nativity	
U.S. born	614 (69.5)
Not U.S. born	256 (29.0)
Missing	14 (1.6)
Education	
< High school	177 (20.0)
High school degree/GED	160 (18.1)
Some college/associates	262 (29.6)
College degree	145 (16.4)
Graduate degree	125 (14.1)
Missing	15 (1.7)
Marital status	
Married	325 (36.8)
Living with partner	262 (29.6)
Single	118 (13.3)
Divorced, separated, widowed	32 (3.6)
Other	135 (15.3)
Missing	12 (1.4)
Employment	
Employed	446 (50.5)
Seeking employment	281 (31.8)
Not seeking employment	138 (15.6)
Missing	19 (2.1)
Finances at end of month	
Not enough money	138 (15.6)
Just enough money	385 (43.6)
Some extra money	342 (38.7)
Missing	19 (2.1)
Financial strain	
Low	216 (24.4)
Moderate	463 (52.4)
High	191 (21.6)
Missing	14 (1.6)
Annual household income	
< $10,000	123 (13.9)
$20,000-$24,999	249 (28.2)
$25,000-$49,999	203 (23.0)
> $49,999	248 (28.1)
Missing	61 (6.9)
Participation in WIC	
No	311 (35.2)
Yes	555 (62.8)
Missing	18 (2.0)

Abbreviations: GED, General Educational Development; WIC, The Special Supplemental Nutrition Program for Women, Infants, and Children

**Table 2:** Fit Statistics, Class Proportions, and Classification Metrics for the one-, two-, and three-class models

	k = 1	k = 2	k = 3
BIC	42,328.44	40,311.53	40,300.21
aBIC	42,074.37	39,800.22	39,531.67
LMR [value; (p-value)]	N/A	2561.79; (p < 0.00)	559.84; (p = 0.37)
VLMR [value; (p-value)]	N/A	2566.45; (p < 0.00)	560.86; (p = 0.36)
BLR [value; (p-value)]	N/A	2561.79; (p < 0.00)	Not trustworthy
Entropy	N/A	0.88	0.89
Mean Posterior Probabilities	N/A	0.96/0.97	0.96/0.94/0.96
Class 1%	100%	35%	38%
Class 2%	N/A	65%	56%
Class 3%	N/A	N/A	5%

**Table 3:** Final CRISYS-SF Items based on the Results of Latent Class Analysis and Expert Consensus*

Domain	Item	Item probabilities		
		Class 1	Class 2	Discrimination
Authority	Did you have trouble with superiors at work?	0.26	0.07	0.19
Career	Did you look for a job?	0.46	0.22	0.24
Career	Did you begin a new job or get promoted?	0.34	0.17	0.17
Financial	Did you miss an appointment or have to change your plans because you had no transportation to get there?	0.4	0.06	0.34
Financial	Did the utility or phone company threaten to cut off your service because you couldn’t pay for it?	0.37	0.06	0.31
Financial*	Did your income decrease by a lot?	0.47	0.23	0.24
Financial*	Did you go without food because you didn’t have the money to pay for it?	0.22	0.02	0.2
Home	Did rats, mice or insects bother you in your home?	0.42	0.22	0.2
Home	Did you lose your housing?	0.14	0	0.14
Legal	Did anyone in your family get arrested?	0.23	0.03	0.2
Legal*	Was anyone in your family pulled over or questioned by the police?	0.21	0.06	0.15
Medical	Did you get admitted to the hospital?	0.33	0.13	0.2
Medical	Did another family member become ill?	0.26	0.13	0.13
Other	Did you have trouble communicating with someone about something that was important to you?	0.27	0.05	0.22
Other	Did your partner ever drink too much or use drugs?	0.25	0.04	0.21
Prejudice	Did someone treat you unfairly because of your age?	0.14	0	0.14
Prejudice	Did someone treat you unfairly because of your race?	0.14	0.01	0.13
Relationships	Did you and your partner disagree about raising your children?	0.39	0.09	0.3
Relationships	Did you and your partner disagree about roles and responsibilities?	0.39	0.1	0.29
Relationships*	Did a family member die?	0.3	0.13	0.16
Relationships*	Did your regular child care arrangements change in any way?	0.25	0.11	0.13
Safety in the Community	Did you hear violence outside your home (e.g., gunfire)?	0.47	0.17	0.3
Safety in the Community	Did you see drug dealing in your building or neighborhood?	0.38	0.1	0.28
Safety in the Home	Did you feel emotionally or physically abused?	0.14	0	0.13

* denotes items that were added based on expert consensus.

See S1 Appendix for a complete list of items used in the latent class analysis in this study.
