# Supplementary material for: A short form of the Crisis in Family Systems (CRISYS) in a racially diverse sample of pregnant women
Source: Curr Psychol. Author manuscript; Available in PMC 2023 May 16. (PMC10182108; doi:10.1007/s12144-021-02335-w)
Supplement: 1804733_Sup_File_2 [file NIHMS1804733-supplement-1804733_Sup_File_2.docx]

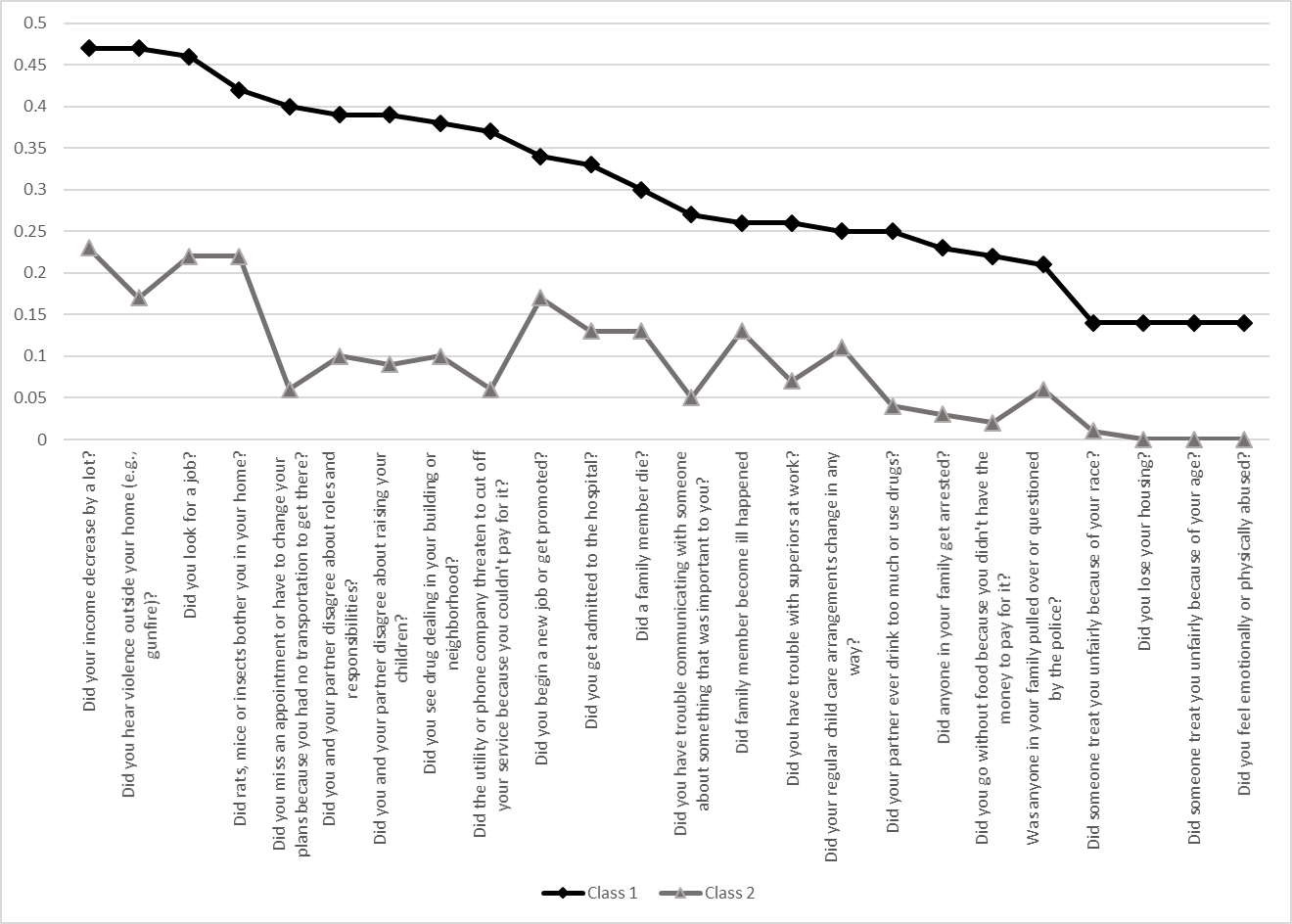
S1 Fig. Expected endorsement probabilities for the CRISYS-RS items chosen from the final latent class model.
