# Supplementary material for: A short form of the Crisis in Family Systems (CRISYS) in a racially diverse sample of pregnant women
Source: Curr Psychol. 2021 Oct 1;42(10):8393–401. doi: 10.1007/s12144-021-02335-w (PMC10182108; doi:10.1007/s12144-021-02335-w)
Supplement: Supplementary file 1 [file 12144_2021_2335_MOESM1_ESM.docx]

**S1 Appendix: Complete list of items used in the construction of the CRISYS-RS.** 

Domain

 

Item

 

Description

 

Authority

 

	19 	Did you trouble with teachers happened 

54 Did you have trouble with social service agencies? 

55 Did you have trouble with medical or health professionals? 

63 	      Did you have trouble with superiors at work? 

Career 

	18 	Did you return to school? 

60 Did you work in the last six months? 

61 Did you begin a new job or get promoted? 

62 Did you get laid off? 

	64 	Did you look for a job? 

Financial 

1 Did your income increase by a lot? 

2 Did you go deeply in debt? 

	3a 	Did your income decrease by a lot? 

	**3b** 	**Did you give money to support family or friends not living with you?** 

4 	     Did you go without food because you didn't have the money to pay for it? 

5 	     Did you go without some clothing because you couldn't pay for it? 

6 Did you miss a rent or mortgage payment because you couldn't pay for it? 

7 Did the utility or phone company threaten to cut off your service because you couldn't pay for it? 

8 Was your telephone, electricity or has turned off? 

9 Did you go without furniture because you didn't have the money to pay for it? 

10 Did you go without an appliance? 

Did you miss an appointment or have to change your plans because you had no transportation to get 

	12 	there? 

Housing 

	11 	Did you lose your housing? 

48 Did a relative or friend move into your home? 

49 Did a relative or friend move out of your home? 

50 Did you move? 

51 Did rats, mice or insects bother you in your home? 

52 Did you have trouble with your landlord? 

53 Did you have trouble with your neighbors? 

Legal 

	13a 	Did you have legal problem? 

	**13b** 	**Did you go without legal advice when you needed it?** 

	**13c** 	**Was anyone in your family pulled over or questioned by the police?** 

	**13d** 	**Were you or your partner questioned about your legal status?** 

	14 	Did anyone in your family get arrested? 

	15a 	Did anyone in your family go to jail? 

Medical (self) 

	37 	Did you(r partner) get pregnant? 

	38a 	Did you(r partner) have a baby? 

39 Did you(r partner have a miscarriage? 

40 Did you(r partner) have an abortion? 

	41a 	Did you ever use alcohol or drugs to get through a day? 

	42 	Did you become ill or did you have a flare up of a chronic illness? 

	**43b** 	**Did you go without medical care when you needed it?** 

44 Did you get admitted to the hospital? 

Medical (others) 

	**38b** 	**Did any of your children (did your child) get pregnant or get someone else pregnant?** 

	**41b** 	**Did partner ever drink too much or use drugs?** 

	43a 	Did your child(ren) become ill or did your child(ren) have a flare up of a chronic illness? 

45 Did your child(ren) get admitted to the hospital? 

46 Did family member become ill happened 

47 Did a friend become ill? 

Prejudice 

56 Did someone treat you unfairly because of your age? 

57 Did someone treat you unfairly because of your sex? 

58 Did someone treat you unfairly because of your race? 

	59a 	Did someone treat you unfairly because you didn't have a lot of money? 

	**59b** 	**Did someone treat you unfairly because of the way you speak?** 

Relationships 

| **15b** | **Did anyone bully your child(ren)?** |
| --- | --- |
| **15c** | **Did child(ren) challenge your family beliefs or values?** |
| **15d** | **Did any of your children (did your child) get bad grades or bad marks in school?** |
| 16a | Did your child(ren) get into trouble? |
| **16b** | **Were any of your children (was your child) involved with someone who you think is a gang member?** |
| 17a | Did you have trouble reading or understanding something that was important to you? |
| **17b** | **Did you have trouble communicating with someone about something that was important to you?** |
| 20 | Did your regular child care arrangements change in any way? |
| 21a | Did you get married? |
| **21b** | **Did you and your partner disagree about raising your children?** |
| **21c** | **Did you and your partner disagree about roles and responsibilities?** |
| **21d** | **Did you miss an important family event that you wanted to attend?** |
| 22 | Did you get a divorce or break up with a partner? |
| 23 | Did you get back together with a partner? |
| 24 | Did a family member die? |
| 25 | Did a friend die? |

 

 

Community 

	26 	Did anything happen in your neighborhood that made you feel unsafe? 

30 Were you a victim of crime while you were outside or away from your home? 

31 Did you hear violence outside your home (e.g., gunfire)? 

32 Did you see violence? 

33 Did your child(ren) see violence? 

34 Was your child (were your children) a victim of a crime? 

35 Was anyone else in your household a victim of a crime? 

36 Did you see drug dealing in your building or neighborhood? 

Home 

27 Did you feel emotionally or physically abused? 

28 Did your child(ren) feel emotionally or physically abused? 

29 Were you a victim of a crime while you were in your home? 

Bolded items are related to acculturation and were added after the publishing of the CRISYS-R. Items with either no suffix or a suffix of ‘a’ were included in the CRISYS-R published by Berry, Shalowitz, Quinn, & Wolf, 2001.
